# Association of the Overall Well-being of a Population With Health Care Spending for People 65 Years of Age or Older

**DOI:** 10.1001/jamanetworkopen.2018.2136

**Published:** 2018-09-14

**Authors:** Carley Riley, Brita Roy, Jeph Herrin, Erica S. Spatz, Anita Arora, Kenneth P. Kell, Elizabeth Y. Rula, Harlan M. Krumholz

**Affiliations:** 1 Department of Pediatrics, University of Cincinnati College of Medicine, Cincinnati, Ohio; 2 Division of Critical Care Medicine, Cincinnati Children’s Hospital Medical Center, Cincinnati, Ohio; 3 Department of Medicine, Yale School of Medicine, New Haven, Connecticut; 4 Health Research and Educational Trust, Chicago, Illinois; 5 Center for Outcomes Research and Evaluation, Yale–New Haven Hospital, New Haven, Connecticut; 6 Section of Cardiovascular Medicine, Department of Internal Medicine, Yale School of Medicine, New Haven, Connecticut; 7 Tivity Health, Franklin, Tennessee; 8 Department of Health Policy and Management, Yale School of Public Health, New Haven, Connecticut

## Abstract

**Importance:**

New US health care payment models have increasingly incentivized health care systems to promote health and reduce health care spending at the population level, with Medicare beneficiaries representing one of the largest populations affected by new payment models. Identifying novel strategies to promote health and reduce health care spending is necessary.

**Objective:**

To assess whether the overall well-being of a population is associated with health care spending for people 65 years of age or older.

**Design, Setting, and Participants:**

This US national, population-based cross-sectional study examined the association between county well-being and Medicare fee-for-service (FFS) spending. Population well-being, a holistic assessment of the overall health of the population comprising interrelated domains, including physical, mental, and social health, as measured by the Gallup-Sharecare Well-Being Index (2010), was linked to the mean spending per Medicare FFS beneficiary (2010) and county characteristics data for all US counties assessed. The data were adjusted for prevalence of 4 low-variation conditions (hip fracture, stroke, colorectal cancer, and acute myocardial infarction) and regional penetration of Medicare Advantage. Data analyses were conducted October 13, 2016, to October 31, 2017.

**Main Outcomes and Measures:**

Mean spending per Medicare FFS beneficiary per county.

**Results:**

In total, 2998 counties were assessed using county-level mean values, with 4 to 7317 participants (mean [SD] number of participants, 755 [1220]) per county. The mean (SD) values of the demographic characteristics of the participants were 50.8% (1.3%) female, 74.9% (16.5%) white, 12.1% (13.0%) black, 4.0% (5.3%) Asian, and 13.7% (14.8%) Hispanic with a mean (SD) of the median county age of 38.2 (4.4) years. Medicare spent a mean (SE) of $992 ($110) less per Medicare FFS beneficiary in counties in the highest quintile of well-being compared with counties in the lowest well-being quintile. This inverse association persisted after accounting for key population characteristics such as median household income and contextual factors such as urbanicity and health care system capacity. Medicare spent a mean (SE) of $1233 ($104) less per Medicare FFS beneficiary in counties with the greatest access to basic needs than in those with the lowest access.

**Conclusions and Relevance:**

In this US national study, the overall well-being of a geographically defined population was inversely associated with its health care spending for people 65 years and older. Identifying this association between well-being and health care spending at the population level may help to lay the foundation for further study to first illuminate the mechanisms underlying the association and to second study interventions aimed at creating greater well-being and lower health care spending at the population level.

## Key Points

**Question:**

Is the overall well-being of a population associated with the health care spending of individuals 65 years of age or older (the highest users of health care services) in that population?

**Findings:**

In this cross-sectional study assessing 2998 US counties, Medicare spent $992 less per fee-for-service beneficiary in counties in the highest quintile of well-being compared with counties in the lowest quintile. This inverse association persisted after accounting for median household income, urbanicity, and health care system capacity.

**Meaning:**

The overall well-being of a geographically defined population was inversely associated with its health care spending for people 65 years or older.

## Teaser

This cross-sectional study uses US county-level well-being data from the Gallup-Sharecare Well-Being Index and annual Medicare fee-for-service spending per beneficiary to assess whether the overall well-being of a population is associated with the health care spending for those 65 years or older.

## Introduction

New US health care payment models have increasingly incentivized health care systems to promote health and reduce health care spending at the population level. Health systems are therefore assuming increasing levels of financial risk and reward for populations, thereby stimulating interest in nonmedical, health-related factors that affect outcomes and spending. Medicare beneficiaries represent one of the largest populations affected by new payment models. In 2017, benefit payments for nearly 60 million Medicare beneficiaries totaled $591 billion, accounting for 15% of the federal budget that totaled $4 trillion.^1^ To manage risk associated with caring for this population, most programs focus on identifying and intervening with high-risk groups.^2,3,4,5,6,7^ A complementary strategy, however, could be targeting areas that might more broadly affect population health and spending.

With prior studies demonstrating an association between well-being and health care use,^8,9,10,11^ the overall well-being of a population may be associated with health care spending and may warrant attention in the efforts to promote the overall health of a population and constrain health care spending. Well-being is a positive state of being beyond the absence of disease, measured by not only physical health but also other dimensions, such as emotional, social, and economic health.^12,13^ Well-being may be modifiable by a broad range of interventions across different sectors. The overall well-being of a population (hereafter referred to as population well-being) reflects the average well-being of individuals within a population^14^ and, as such, can convey information about the population different from what can be gleaned from individual-level data alone. It is plausible that the well-being of a population is associated with the health care spending within that population through a myriad of mechanisms, such as those related to economic factors, the social environment, and the health-related behaviors of the population.

To assess whether well-being and health care spending are correlated across counties, we used county-level well-being data from the Gallup-Sharecare Well-Being Index (WBI), previously known as the Gallup-Healthways WBI prior to rebranding following Sharecare’s 2016 acquisition of Healthways (Gallup-Sharecare, 2010), and annual Medicare fee-for-service (FFS) spending per beneficiary (Dartmouth Atlas, 2010). We focused on spending for the Medicare FFS population in part because it may be less sensitive to individual resources, more uniform nationally, and driven more by use than price variation in comparison with spending within Medicare Advantage (MA) or private insurance plans.^15^ We hypothesized that expenditures per Medicare FFS beneficiary would be lower among beneficiaries in counties with a higher overall well-being, even after accounting for other area-level factors that might be associated with health care spending.

## Methods

### Overview

We examined the cross-sectional association between population well-being and Medicare spending at the county level.^16^ We linked survey measures of well-being to mean spending per Medicare FFS beneficiary and county characteristics data for all counties in the United States. We used weighted regression models to assess independent associations of spending with population well-being after accounting for county characteristics, including rates of chronic conditions, urban setting, median household income, and health care system capacity. We also examined the association between well-being and spending in counties stratified by urbanicity, household income, and health care system capacity. Finally, we used weighted regression models to assess independent associations of spending with each domain of well-being. The Yale University institutional review board approved this study and waived the need for obtaining informed participant consent. We followed the Strengthening the Reporting of Observational Studies in Epidemiology (STROBE) reporting guideline.

### Health Care Spending

Our primary outcome was mean Medicare spending per beneficiary at the county level. We used 2010 values from the Dartmouth Atlas based on reimbursements for all FFS beneficiaries enrolled in Medicare Parts A and B, risk standardized for population age, sex, and race/ethnicity and adjusted for the Medicare price schedule.^17^

### Population Well-being

Our primary independent variable was population well-being, as measured by the Gallup-Sharecare WBI (2010).^18^ The WBI was developed based on the work of experts in psychology and has acceptable reliability and internal and external validity.^19,20,21^ Since 2008, Gallup has surveyed a unique sample of nearly 1000 individuals 18 years of age or older every day, approximately 350 days per year. Stratified random sampling is used with respondents surveyed from all 50 states and the District of Columbia. The survey is administered in English and Spanish, using landline telephones and cell phones, and comprises 55 self-reported items organized into 6 domains: life evaluation (life satisfaction and optimism), emotional health (daily emotions and the presence or absence of depression), physical health (chronic disease and recent illness), healthy behaviors (smoking, exercise, and fruit or vegetable consumption), basic access (perceived safety, housing, and health care access), and work environment (job satisfaction and trust or respect in the workplace for respondents who report being employed). Each domain is scored separately. A composite score, the WBI, is calculated as the unweighted mean of the domain scores. All are reported on a scale of 0 to 100, where the higher the score the higher one’s well-being; these individual scores were aggregated at the county level to produce WBI and domain scores.

### Other Independent Variables

We used Medicare FFS inpatient claims data (2010) to calculate county rates (per Medicare FFS beneficiary) of hospital admissions for 4 low-variation conditions (LVCs), included as a clinical risk adjuster consistent with the method used by Colla et al^22^ in their study of variation in health care spending: hip fracture, stroke, colorectal cancer, and acute myocardial infarction. These LVCs require acute care hospitalization, are less subject to diagnostic intensity or coding practices, are therefore reflective of the true disease burden for these conditions, and have been shown to be associated with regional mortality and health care expenditures.^22^ To construct this variable, we used individual-level data from Medicare beneficiaries who were aged 64 years or older, enrolled in FFS for at least 1 month, and discharged from a short-term, acute-care hospital in the United States between January 1 and December 31, 2010. We linked beneficiaries to a county based on the residence postal code variable in the Medicare database. All LVC indicators were used to calculate rates at the county level, and counties were categorized into quintiles of each rate for analysis. Because the proportion of Medicare beneficiaries enrolled in MA plans (MA penetration) varies by income, which may in turn affect health care use, we also adjusted each model for MA penetration.^23^

In additional variations of our model, as described herein, we accounted for the following county characteristics (using the Area Health Resource File), which we hypothesized could affect the association between well-being and health care spending: median age, percentage of each sex, percentage of each racial/ethnic category, percentage of urban residents, median household income, and health care system capacity variables (general practitioners per capita, specialists per capita, hospital beds per capita, and full-time physicians and dentists). We classified counties using Area Health Resource File urban continuum categories (metropolitan vs other). We also constructed a single health care system capacity variable from the 4 health care system capacity variables listed; this was calculated by first standardizing each variable to have a mean (SD) value of 0 (1), and then averaging the 4 standardized variables.

### Statistical Analysis

We summarized spending and independent variables by WBI quintile, reporting mean values and SDs for each quintile. To examine the association between county-level well-being and beneficiary Medicare spending, we estimated a series of regression models, each using county mean per beneficiary Medicare spending as the dependent variable. Because county spending distribution was approximately normal, we used linear models. All linear regressions were weighted by number of WBI respondents per county. Model 1 included quintiles of WBI with the lowest quintile omitted and used as the reference, the LVC variables, and MA penetration. Subsequent models extended model 1 to include percentage of urban residents (model 2); median household income (model 3); the 4 health care system capacity variables (model 4a); composite health care system capacity metric (model 4b); and percentage of urban residents, household income, and the 4 health care system capacity variables (model 5). We ran multiple models in which we added variables sequentially to assess the influence of different factors on the association. For each model, we used Wald *P* values to test for an overall WBI association.

We performed several secondary analyses. We tested for interactions with WBI and urbanicity, household income, and health care system capacity; to aid interpretation of the interaction term, we entered WBI as a continuous variable rather than as a set of quintile indicators. For significant interactions, we replicated the main model within each strata of household income (tertiles) and health care system capacity (above or below national mean). Finally, we replicated the models using each of the domains, categorized into quintiles rather than overall WBI as the independent variables, to better understand how different dimensions of well-being contribute to the association of overall well-being and spending. All analyses were performed from October 13, 2016, to October 31, 2017, using Stata, version 15.0 (StataCorp). A 2-sided *P* < .05 was considered statistically significant.

## Results

### Descriptive Results

We used data from 2998 counties with survey responses from the Gallup-Sharecare WBI database. The mean (SD) number of participants per county was 755 (1220) (range, 4-7317). The county-level mean values of the demographic characteristics of participants are reported in Table 1. The mean (SD) values of the demographic characteristics of the participants were 50.8% (1.3%) female, 74.9% (16.5%) white, 12.1% (13.0%) black, 4.0% (5.3%) Asian, and 13.7% (14.8%) Hispanic, with a mean (SD) of the median county age of 38.2 (4.4) years. The mean (SD) county-level WBI score was 67.8 (3.2) (range, 39.9-90.6). County Medicare spending per beneficiary ranged from $5374 to $15 967 (mean [SD], $9660 [$1334]). There was statistically significant variation in all covariates across well-being quintiles (Table 1).

**Table 1.** Demographic and Health Care System Capacity Characteristics for All Counties and by Quintile of Composite Gallup-Sharecare 2010 WBI Score^a^

Characteristic	Mean (SD) Value (n = 2998)	WBI Quintile^b^				
		1 (n = 599)	2 (n = 597)	3 (n = 613)	4 (n = 588)	5 (n = 580)
No. of participants per county	755 (1220)	50 (38)	358 (481)	757 (952)	1130 (1758)	635 (655)
Composite WBI score	67.8 (3.2)	59.8 (3.0)	64.8 (0.8)	67.0 (0.6)	69.1 (0.7)	71.9 (1.5)
Age, median (SD), y	38.2 (4.4)	40.6 (3.6)	38.9 (4.1)	37.8 (4.2)	38.0 (4.7)	38.0 (4.3)
Sex, %						
Male	49.2 (1.3)	49.8 (1.9)	49.3 (1.5)	49.1 (1.2)	49.1 (1.0)	49.3 (1.1)
Female	50.8 (1.3)	50.2 (1.9)	50.7 (1.5)	50.9 (1.2)	50.9 (1.0)	50.7 (1.1)
Race/ethnicity, %						
White	74.9 (16.5)	83.2 (17.1)	77.0 (17.8)	73.0 (16.1)	74.2 (15.3)	74.6 (16.7)
Black	12.1 (13.0)	10.1 (15.3)	13.4 (14.9)	13.6 (12.7)	11.8 (12.1)	9.5 (11.9)
Asian	4.0 (5.3)	0.5 (0.4)	1.8 (2.1)	3.6 (4.4)	4.2 (4.4)	7.2 (8.5)
Hispanic or Latino	13.7 (14.8)	6.1 (12.3)	10.4 (13.0)	16.0 (16.1)	15.7 (15.7)	11.8 (11.0)
Other	5.2 (5.6)	2.4 (4.4)	4.2 (5.5)	5.9 (5.7)	6.0 (6.1)	4.6 (4.1)
Urban, %	7.6 (2.7)	3.4 (2.2)	6.5 (2.8)	8.0 (2.3)	8.3 (2.1)	8.0 (2.6)
Household income, × $1000	39.4 (11.1)	29.4 (4.3)	32.3 (4.4)	37.2 (7.4)	41.9 (11.3)	47.7 (13.6)
Health care system capacity, No. per 100 000 population						
General practitioners	29.7 (15.3)	24.5 (15.1)	27.0 (15.1)	27.9 (14.9)	30.4 (13.8)	35.2 (17.0)
Specialists	90.5 (76.1)	23.0 (23.1)	56.5 (45.0)	87.9 (65.7)	103.4 (76.6)	122.3 (95.7)
Hospital beds	0.3 (0.2)	0.3 (0.4)	0.3 (0.3)	0.3 (0.2)	0.3 (0.2)	0.3 (0.2)
Full-time equivalents of physicians or dentists	37.9 (59.3)	25.7 (45.2)	36.5 (48.5)	42.8 (65.5)	39.7 (65.6)	32.2 (46.8)
Health care system capacity score, normalized	0.2 (0.6)	−0.2 (0.4)	0.1 (0.5)	0.2 (0.6)	0.3 (0.6)	0.4 (0.6)
Score in counties with low capacity	1848.0 (61.6)	453.0 (15.1)	415.0 (13.8)	356.0 (11.9)	311.0 (10.4)	297.0 (9.9
Score in counties with high capacity	1150.0 (38.4)	146.0 (4.9)	182.0 (6.1)	257.0 (8.6)	277.0 (9.2)	283.0 (9.4)
Total Medicare per enrollee reimbursement (SD), $	9660 (1334)	10 157 (1409)	10 012 (1305)	9928 (1282)	9594 (1284)	8891 (1159)

Abbreviation: WBI, Well-Being Index.

^a^ Gallup-Sharecare WBI was previously known as the Gallup-Healthways WBI prior to rebranding following Sharecare’s 2016 acquisition of Healthways.

^b^ *P* < .001 for all.

### Adjusted Results

All models found a significant inverse association between county-level WBI and annual Medicare FFS spending per beneficiary (Table 2). In the model that adjusted for LVCs only (model 1), people in counties in the highest quintile of well-being scores cost the government a mean (SE) of $920 ($108) less per Medicare FFS beneficiary per year than counties in the lowest quintile. In the models that additionally adjusted for percentage of urban residents (model 2) and household income (model 3), people in the highest well-being counties cost a mean (SE) of $1195 ($110) and $1060 ($112) less per Medicare beneficiary, respectively, than the lowest well-being counties, indicating that these variables augmented rather than attenuated associations. By contrast, adjusting for health care system capacity resulted in a mean (SE) difference of $836 ($108) in spending between the highest and lowest well-being counties (model 4). The fully adjusted model (model 5) indicated that after adjusting for LVCs, MA penetration, percentage of urban residents, household income, and health care system capacity, the highest well-being counties spent a mean (SE) of $992 ($110) less in Medicare spending per beneficiary than the lowest well-being counties (*P* < .001) (Figure). In sensitivity analyses, excluding counties with less than 100 respondents did not substantially change the results.

**Table 2.** County-Level Associations Between Composite Gallup-Sharecare 2010 WBI Score and Mean Annual Medicare Spending per Enrollee^a^

WBI Score Quintile	Difference in Medicare Spending/Enrollee, Mean (SE), $^b^	*R*^2^ Value
Model 1^c^		
1	1 [Reference]	0.28
2	−367.4 (104.5)	
3	−399.2 (100.7)	
4	−582.7 (101.3)	
5	−920.3 (107.6)	
Model 2^d^		
1	1 [Reference]	0.30
2	−549.7 (104.9)	
3	−673.9 (103.7)	
4	−861.3 (104.4)	
5	−1194.5 (110.3)	
Model 3^e^		
1	1 [Reference]	0.28
2	−387.7 (104.2)	
3	−447.4 (100.9)	
4	−671.5 (102.9)	
5	−1059.7 (111.7)	
Model 4a^f^		
1	1 [Reference]	0.34
2	−315.1 (100.2)	
3	−337.1 (97.1)	
4	−507.8 (98.5)	
5	−812.1 (106.9)	
Model 4b^g^		
1	1 [Reference]	0.29
2	−334.2 (104.1)	
3	−342.3 (100.7)	
4	−518.1 (101.5)	
5	−836.1 (108.2)	
Model 5^h^		
1	1 [Reference]	0.35
2	−470.8 (101.0)	
3	−560.3 (100.0)	
4	−719.0 (101.4)	
5	−991.6 (109.8)	

Abbreviations: LVC, low-variation condition; MA, Medicare Advantage; WBI, Well-Being Index.

^a^ Gallup-Sharecare WBI was previously known as the Gallup-Healthways WBI prior to rebranding following Sharecare’s 2016 acquisition of Healthways.

^b^ *P* < .001 for all.

^c^ Base model adjusted for MA penetration and LVCs.

^d^ Base model adjusted for MA penetration, LVCs, and percentage of urban residents.

^e^ Base model adjusted for MA penetration, LVCs, and median household income.

^f^ Base model adjusted for MA penetration, LVCs, and 4 variables of health care system capacity.

^g^ Base model adjusted for MA penetration, LVCs, and health care system capacity score.

^h^ Model 5, base model adjusted for MA penetration, LVCs, percentage of urban residents, median household income, and 4 variables of health care system capacity.

**Figure.**
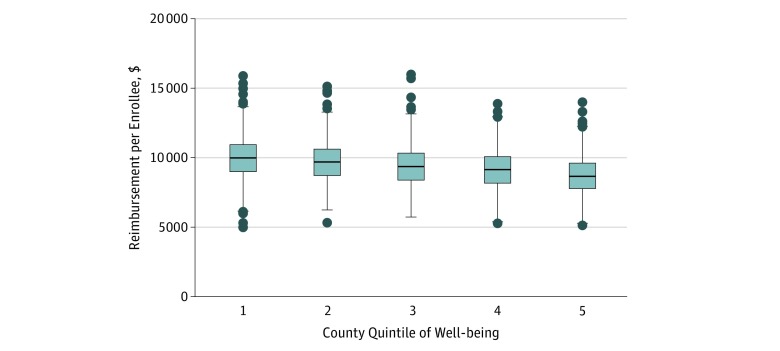
Reimbursement per Enrollee for Medicare Fee-for-Service by County Quintile of Well-being The horizontal line in the middle of each box indicates the median, whereas the top and bottom borders mark 75th and 25th percentiles, respectively. The whiskers above and below each box indicate the 90th and 10th percentiles; the points beyond the whiskers indicate outliers beyond the 90th and 10th percentiles.

### Interactions and Stratified Results

Interactions with income (mean [SE], −3.2 [0.7]; *P* < .001) and health care system capacity (−46.1 [12.0]; *P* < .001) were significant, but that with urbanicity was not (−3.0 [2.1]; *P* = .15). Thus, we performed 2 stratified analyses using fully adjusted models. All stratified analyses revealed differences in the association between population well-being and Medicare spending across the examined strata, but the inverse association was maintained in all 4 strata and models (*P* < .001 for all) (Table 3). The mean (SE) difference in Medicare spending per beneficiary between the highest and lowest well-being quintiles was greater in counties with higher household income (−1198.5 [358.0] vs −508.2 [183.5]) and with greater health care system capacity (−1184.1 [243.8] vs −669.1 [116.3]).

**Table 3.** County-Level Associations Between Composite WBI Score and Mean Annual Medicare Spending per Enrollee, Stratified by Percentage of Urban Residents, Median Household Income, and Health Care System Capacity Score, From Fully Adjusted Model^a^^,^^b^

WBI Score Quintile	Difference in Medicare Spending/Enrollee, Mean (SE), $	*P* Value	*R*^2^ Value
Model 5			
1	1 [Reference]	<.001	0.3537
2	−470.8 (101.0)		
3	−560.3 (100.0)		
4	−719.0 (101.4)		
5	−991.6 (109.8)		
Stratified by median household income			
High			
1	1 [Reference]	<.001	0.4232
2	−463.0 (363.5)		
3	−811.0 (356.1)		
4	−897.1 (354.4)		
5	−1198.5 (358.0)		
Middle			
1	1 [Reference]	<.001	0.3933
2	−621.4 (149.4)		
3	−560.1 (149.0)		
4	−849.4 (156.9)		
5	−785.0 (191.1)		
Low			
1	1 [Reference]	.09	0.2324
2	−146.3 (117.9)		
3	−180.4 (129.5)		
4	−202.4 (145.1)		
5	−508.2 (183.5)		
Stratified by health care system capacity score			
High			
1	1 [Reference]	<.001	0.4126
2	−556.7 (237.4)		
3	−551.0 (232.5)		
4	−843.9 (234.0)		
5	−1184.1 (243.8)		
Low			
1	1 [Reference]	<.001	0.2672
2	−423.4 (94.8)		
3	−648.6 (96.6)		
4	−564.9 (100.7)		
5	−669.1 (116.3)		

Abbreviation: WBI, Well-Being Index.

^a^ Gallup-Sharecare WBI was previously known as the Gallup-Healthways WBI prior to rebranding following Sharecare’s 2016 acquisition of Healthways.

^b^ This is model 5, which is the base model adjusted for Medicare Advantage penetration, low-variation conditions, percentage of urban residents, median household income, and 4 variables of health care system capacity.

### Domain Results

Fully adjusted models assessing independent associations between quintiles of each well-being domain and health care spending are reported in Table 4. Similar to the composite WBI, each domain showed lower spending per Medicare beneficiary with increasing county-level domain score. The greatest difference in spending occurred with the basic access index, one of the domains of the WBI that includes access to health care, clean water, fresh produce, and safe public space, as well as ability to afford basic needs such as food and shelter. Medicare spent a mean (SD) of $1233 ($104) less per Medicare FFS beneficiary in counties with the highest basic access scores than in counties with the lowest basic access scores. The next 3 greatest differences, in order of decreasing magnitude, occurred with the healthy behaviors, emotional health, and physical health indices.

**Table 4.** Fully Adjusted County-Level Associations Between Gallup-Sharecare 2010 WBI Domain Scores and Mean Annual Medicare Spending per Enrollee, From the Fully Adjusted Model^a^^,^^b^

WBI Score Quintile	Difference in Medicare Spending/Enrollee, Mean (SE), $^c^	*R*^2^ Value
Physical health		
1	1 [Reference]	0.3507
2	−693.2 (100.1)	
3	−752.6 (97.9)	
4	−566.3 (100.4)	
5	−793.0 (110.8)	
Emotional health		
1	1 [Reference]	0.3575
2	−382.0 (86.7)	
3	−715.7 (87.1)	
4	−723.2 (89.8)	
5	−808.1 (108.9)	
Life evaluation		
1	1 [Reference]	0.3437
2	−470.5 (106.3)	
3	−393.1 (104.4)	
4	−292.5 (105.6)	
5	−594.5 (111.1)	
Healthy behaviors		
1	1 [Reference]	0.3609
2	−242.4 (88.2)	
3	−516.6 (86.9)	
4	−732.3 (88.7)	
5	−868.5 (98.2)	
Work environment		
1	1 [Reference]	0.3425
2	−132.4 (91.7)	
3	−336.7 (92.6)	
4	−432.1 (93.1)	
5	−174.7 (112.2)	
Basic access		
1	1 [Reference]	0.3756
2	−529.6 (97.4)	
3	−650.7 (95.2)	
4	−1048.4 (96.6)	
5	−1233.4 (103.5)	

Abbreviation: WBI, Well-Being Index.

^a^ Gallup-Sharecare WBI was previously known as the Gallup-Healthways WBI prior to rebranding following Sharecare’s 2016 acquisition of Healthways.

^b^ This is model 5, which is the base model adjusted for Medicare Advantage penetration, low-variation conditions, percentage of urban residents, median household income, and 4 variables of health care system capacity.

^c^ *P* < .001 for all.

## Discussion

We found that higher county well-being was associated with lower health care spending per Medicare FFS beneficiary. This association between population well-being and spending was independent of factors that could confound the association, such as urbanicity, median household income, and health care system capacity. Within strata of household income and health care system capacity, the inverse association between well-being and Medicare dollars spent remained consistent but was considerably stronger in counties with higher household income and greater health care system capacity. In addition, well-being domain analyses revealed that all domain scores exhibited an inverse association with spending, with the basic access index score demonstrating the strongest association, followed by the scores for the emotional health, healthy behaviors, and physical health indices.

Our study used a rigorously developed, multidimensional, survey-based assessment of well-being from a national sample to extend the literature by examining how health care spending of a particular subpopulation is associated with the overall adult well-being within the population as a whole. Prior literature has mostly examined the association between health care use, the leading driver of spending in the Medicare population, and well-being and has largely done so at the individual level.^8,9,10,11,15^ A few studies have found that individual well-being is inversely associated with individual health care use and costs in privately insured populations,^8,10,11^ whereas an additional study found that well-being in individuals older than 64 years is associated with lower levels of disease prevalence and use in the Medicare FFS population.^9^ The present study adds to the existing literature by examining population-level associations between well-being and health care spending. This population-level analysis of the association between mean well-being of residents and county-level spending on Medicare FFS beneficiaries allows for an assessment of associations at the group level that may be different from associations at the individual level. Studying this association at the county level allows us to assess ecological and community-level contributions. With our county-level assessment, we also could account for potential confounding across key population and contextual characteristics, such as urbanicity, median household income, and local health care system capacity. In addition, the county is a relevant geographic unit because policies and programs are often enacted at the county level.

There are several explanations for why higher mean adult well-being within a county may be associated with lower health care spending for its Medicare FFS subpopulation. It may be that behaviors and emotional states spread from person to person in a population,^24,25,26^ such that living among people with higher well-being can confer health-related benefits that lead to lower health care spending. At the individual level, higher well-being is associated with healthier behaviors, such as not smoking, exercising regularly, and achieving better sleep,^27,28,29^ and healthier physiological attributes, such as greater heart rate variability, lower blood pressure levels, lower levels of inflammatory factors, and lower cortisol levels.^30,31,32,33,34,35^ At the population level, higher mean well-being is associated with better population health outcomes, such as longer life expectancy and lower mortality rates.^36^ If living in a higher well-being population fosters healthier behaviors and physiological attributes, populations with higher well-being could require lower health care spending.

It may also be that higher well-being populations either create or result from positive environmental and social conditions that confer benefits that result in lower health care spending. Higher well-being populations exhibit higher levels of positive psychosocial factors, such as trust, social support, social cohesion (defined as social ties among community members^37^), and social capital (defined as social networks, norms, and trust that support cooperation for mutual benefit^38^), each of which is associated with better health outcomes in general and for older adults in particular.^13,39,40,41,42,43,44,45,46,47,48,49^ As such, with higher levels of these social assets, higher well-being populations could achieve better population health with less health care spending. Through direct effects on health status and indirect effects by which social support protects against the pathogenic influence of stress, social support encourages health.^50^ For individuals with coronary heart disease, social support has been associated with lower risk of primary and secondary events,^51^ lower mortality,^52^ and better compliance with rehabilitation programs.^53^ Social support has also been associated with better outcomes in other health conditions.^54,55,56,57^ Conversely, social isolation or nonsupportive social interaction has been associated with worse cardiovascular, endocrine, and immune system pathophysiology^58,59,60^ as well as higher health care use and cost of care.^61^ In addition, a population with higher social capital may be able to better leverage social support to maintain elderly people in the home or community setting than another population with lesser social capital and thus potentially greater reliance on the health care system.^38^

The results of the present study could have several potential policy implications. Although the associations among well-being, its different domains, and health care spending are complex, and the mechanisms underlying the association are not yet identified, our findings suggest the possibility that certain population-level investments may not only increase well-being, a laudable goal itself, but also result in reduced health care spending. With nearly $1000 less in spending per Medicare FFS beneficiary in the highest well-being counties compared with the lowest well-being counties, a 10% difference in spending, the return on investment for programs and policies that improve well-being and lower health care spending is potentially substantial. Based on the well-being domain results, interventions that improve basic access for populations may yield substantial improvements in both overall well-being and health care spending. Currently in many nations, including the United States, efforts to improve population well-being are under way.^62,63,64,65,66,67^ Some interventions involve multisector, community-based programs, many government supported, while others include economic and social policy changes, such as those aimed at housing, employment, and access to public spaces.^13,26,68,69^ Given the association between population well-being and health care spending, examining how programs and policies influence not only well-being but also health care spending would be valuable and allow for increasing the scale and the spread of interventions that effectively increase well-being and reduce health care spending.

Although our study showed an association between population well-being and health care spending, many questions remain, including questions about what aspects of population well-being are most strongly associated with, and potentially driving, health care spending. The results of our domain analyses are exploratory but provide information about which aspects of well-being are most strongly associated with variation in spending. Following county-level basic access, healthy behaviors and emotional health were associated most strongly with lower Medicare spending. The physical health domain, which captures physical and mental well-being, was also associated with lower spending, although to a lesser extent, potentially as a result of controlling for certain physical health factors with the inclusion of the clinical risk adjuster. Collectively, these findings suggest the possibility that investments in well-being beyond those in physical and mental health alone may be valuable to well-being and health care spending, a possibility that warrants further study. In addition, the stratified analyses, showing that the inverse association between overall well-being and Medicare spending was stronger in higher income counties, suggest that population well-being may have a greater effect on its health care spending in settings that are not confronting the challenges of a lower-resource setting. It may be that factors responsible for Medicare spending other than population well-being are more influential than overall well-being in counties with lesser resources. The domain analyses that showed a strong association between a county’s basic access and its Medicare spending support this theory. Perhaps, for example, access-related factors are so dominant in lower-resource settings that the other domains, and therefore overall well-being, are not as strongly associated with spending in lower-resource settings. Together, these results suggest that different community characteristics—and different aspects of population well-being—may influence health care spending differently, depending on the context, including income and access to resources, within which the population lives. Because well-being is modifiable, these findings raise the possibility that interventions to improve population well-being could lead to lower health care spending.

### Limitations

Our study has several limitations. First, as a cross-sectional study, it cannot demonstrate causation. However, determining whether an association between population well-being and health care spending exists is an essential first step. A second, related limitation is the potential for endogeneity, particularly with physical health as a component of well-being. To address this, we included low-variation conditions to account for burden of chronic disease within each county. Moreover, our separate domain analyses confirmed that, although physical health and spending were associated, other components of well-being, especially basic access, emotional health, and healthy behaviors, were also strongly and inversely associated with spending. Third, the data for this study are from 2010, the most recent year with complete WBI and Medicare FFS spending data at the time we acquired data. Although the data were 8 years old at time of publication, the associations identified are still highly relevant. Given the increasing interest in both well-being as a broader construct of health and innovative strategies to address spending on health care, the present study provides a valuable addition to the current literature. To our knowledge, there is no reason to believe that the association described here would have changed with time. Fourth, by limiting our health care spending data to the Medicare FFS population, we were unable to assess the association between population well-being and health care spending in other insured and uninsured populations. Given the results of a recent study that showed that Medicare payment was largely driven by use, whereas spending for the privately insured was driven largely by price variation,^15^ it is plausible that the association between population well-being and health care spending differs between these 2 populations. Nonetheless, assessing the association between well-being and Medicare FFS spending, particularly given the considerable resources used within Medicare FFS, is valuable. Finally, the WBI sampling method is designed to be representative at state and congressional district levels but not at the county level.

## Conclusions

In this national study, the overall well-being of a geographically defined population was inversely associated with its health care spending for people 65 years of age or older. Identifying this association between well-being and health care spending at the population level lays the foundation for further study to illuminate the mechanisms underlying the association and for subsequent study of interventions aimed at creating higher well-being and lower health care spending in the population.
